# Employing zebrafish to understand genetic drivers of epilepsy-related comorbid behaviors

**DOI:** 10.3389/fphar.2026.1781517

**Published:** 2026-05-04

**Authors:** Chinwendu Ononuju, Kharma Hall, Olivia Beatty, Scott C. Baraban, Colleen Carpenter-Swanson

**Affiliations:** 1 Epilepsy Research Laboratory and Weill Institute for Neuroscience, Department of Neurological Surgery, University of California San Francisco, San Francisco, CA, United States; 2 Department of Biology, University of Richmond, Richmond, VA, United States

**Keywords:** behavioral phenotypes, clemizole, comorbidities, disease modeling, epilepsy, zebrafish

## Abstract

Children with epilepsy frequently experience a range of significant comorbidities beyond seizures, such as motor dysfunction, cognitive impairment, and neurodevelopmental delays. In some cases, these comorbidities contribute more significantly to overall disease burden than the seizures themselves. To improve quality-of-life (QOL) for these children, treatment options should be selected that control seizures and ameliorate comorbidities. Unfortunately, such therapeutics remain largely elusive. Addressing this issue requires suitable preclinical models. Here we used CRISPR-generated zebrafish with single-gene mutations linked to pediatric epilepsy and applied clinically-relevant behavioral assays to study the effects of these mutations. Using high-throughput locomotion-based assays, we uncovered errors in sensorimotor integration in larval *scn1lab, stxbp1b*, *arxa* and *gabrb3* zebrafish mutants at 6 days post-fertilization (dpf) compared to wild-type sibling controls. Strikingly abnormal exploratory and preference responses were also observed in *scn1lab* and *stxbp1b* zebrafish mutants. Pharmacological testing revealed that, compared to stiripentol and valproic acid, clemizole produced the most extensive rescue of deficits in *scn1lab* mutants. Within the broader epilepsy research landscape, this study further supports the use of zebrafish as a robust, dual platform to understand and discover novel therapeutics for epilepsy and its associated behavioral comorbidities.

## Introduction

Epilepsy is an umbrella term for debilitating brain disorders marked by spontaneous, recurrent seizures and is associated with a markedly increased risk of premature mortality, including sudden unexpected death in epilepsy (SUDEP) (Ficker, 2000; Fisher et al., 2005; Nickels et al., 2012). These disorders afflict up to 1% of the global pediatric population, making it the most frequent long-term neurologic condition observed in children (Shinnar and Pellock, 2002; Bastos and Cross, 2020); a third of these patients present with pharmaco-resistant seizures (Arhan et al., 2010; Kwan et al., 2010). Epilepsy is also associated with comorbid disorders (such as autism spectrum disorder), and pediatric patients may exhibit one or more of these conditions that can arise prior to, during or after seizure onset (Gaitatzis et al., 2004; Hamiwka and Wirrell, 2009; Noebels, 2015). These comorbidities can be up to eight times more prevalent in children with epilepsy compared to the general population and are a mounting concern as they negatively affect epilepsy prognosis, reduce QOL and create significant financial and emotional burden for the families (Bazil, 2004; Wiebe and Hesdorffer, 2007; Seidenberg et al., 2009; Russ et al., 2012; Wood, 2012; Keezer et al., 2016). For example, comorbidities can lead to poor seizure outcome (Keezer et a., 2016) and it is estimated that nearly 80% of direct medical costs charged to persons with epilepsy are consequences of comorbid manifestations versus epilepsy itself (Ivanova et al., 2010; Keezer et al., 2016; Olschewski et al., 2019). While more effective antiseizure medications are needed, managing childhood epilepsy also requires addressing any coexisting comorbid conditions.

Comorbidities associated with epilepsy can be classified as psychiatric (e.g., anxiety, autism spectrum disorder, attention-deficit/hyperactivity disorder and depression) or somatic (e.g., migraine, motor dysfunction and sensory impairments), the latter referring to systemic and neurological diseases (Shorvon, 2014). The relationship between epilepsy and its comorbidities may be driven by one or more mechanisms, including shared risk factors, causative interactions (direct and indirect) and bidirectional effects (Gaitatzis et al., 2012; Keezer et al., 2016; Olschewski et al., 2019). Within the subcategory of shared risk factors, there is growing interest in the interplay between genetics, epilepsy and its associated disorders. GWAS and next-generation sequencing provide an ever-growing list of single gene *de novo* mutations that have been found in pediatric epilepsy patients (Mastrangelo and Leuzzi, 2012; Allen et al., 2013; Abou-Khalil et al., 2018; Liu et al., 2018; Fernández-Marmiesse et al., 2019) and understanding the pathogenic function of these genes is critical.

Zebrafish are a powerful model system to uncover developmental and behavioral functions of these human genes (Haesemeyer and Schier, 2015; Ijaz and Hoffman, 2016; Mishra and Nayak, 2025) and have evolved as an impressive tool for studying epilepsy (Hortopan et al., 2010; Griffin et al., 2018; Gawel et al., 2020; D'Amora et al., 2023). By 3 dpf, electrical recordings of spontaneous and induced seizures can be captured from the larval brain, a technique first developed in the Baraban laboratory (Baraban et al., 2005; Baraban, 2013). The optical transparency during embryonic and early larval stages allows for detailed brain imaging studies in live animals. For example, whole brain calcium imaging studies have shown the dynamic neural events that occur during seizures (Turrini et al., 2017; Liu and Baraban, 2019; Hadjiabadi et al., 2021; Liu et al., 2021; de Vito et al., 2022; Hasani et al., 2023). Zebrafish fecundity and ability of larvae to fit in 96- and 384- well formats also offer many advantages for high-throughput drug screening (Chakraborty et al., 2009). For example, serotonin receptor modulating drugs lorcaserin and clemizole were initially identified as exhibiting antiseizure activity in the *scn1lab* zebrafish model of Dravet syndrome (Baraban et al., 2013; Griffin et al., 2017) before moving into clinical trials for this patient population. Furthermore, zebrafish provide an alternative system to conventional rodent models for studying complex behavioral paradigms such as learning and social interactions (Geng and Peterson, 2019; Tan et al., 2022).

We previously generated CRISPR-edited zebrafish models to elucidate the causative roles of 40 loss-of-function (LOF) mutations associated with childhood encephalopathies. Epilepsy phenotypes in these lines were rigorously assessed using electrophysiological recordings, revealing that approximately 20% exhibit spontaneous electrographic seizures (Grone et al., 2016; Griffin et al., 2021). Genes identified among these ‘seizure-positive’ lines span several functional categories, including transcriptional regulation of cortical development (*arxa*), protein translation and neuronal maintenance (*eef1a2*), inhibitory neurotransmission (*gabrb3*), glutamatergic receptor signaling (*grin1b*), vitamin B6 metabolism (*pnpo*), voltage-gated sodium channel function (*scn1lab*), mTOR pathway signaling (*strada*), and synaptic vesicle release (*stxbp1b*). Here, we employed six of these models (*arxa, eef1a2, gabrb3, scn1lab, strada,* and *stxbp1b*) to further investigate the relationship between LOF mutations and the development of clinically relevant behavioral comorbidities in the context of epilepsy. We examined homozygous mutants 6–7 days after fertilization using startle and light-dark preference assays to measure motor responses, learning, and exploratory behaviors, and often observed differences in these larvae compared to their wild type siblings. Preference tests were conducted using a custom chamber optimized for locomotion-based video tracking on a DanioVision system (Noldus Information Technology). We viewed these aberrant behaviors as surrogate markers for behavioral comorbidities and using pharmacological approaches, investigated whether we could ameliorate these phenotypes.

## Materials and methods

### Zebrafish maintenance

Zebrafish were maintained in a temperature-controlled facility on a 14/10 h light-dark cycle, with lights on at 9:00 a.m. and off at 11:00 p.m. Experimental procedures were in accordance with the Guide for the Care and Use of Animals (eLibrary Inc, 2011) and the guidelines followed were approved by the University of California, San Francisco Institutional Animal Care and Use Committee (IACUC approval #: AN171512-03) and the University of Richmond Institutional Animal Care and Use Committee (IACUC approval #: 21-05-001). Male and female adult zebrafish were housed on aquatic units with an automated feedback control unit that maintained the system water conditions within the following ranges: temperature, 28–30 °C; pH, 7.5–8.0 and conductivity, 690-740 μS/cm. Zebrafish embryos and larvae were raised in an incubator kept at 28.5 °C under the same light-dark cycle as the facility. The solution or ‘embryo medium’ used for the embryos and larvae consisted of 0.03% Instant Ocean (Aquarium Systems, Inc.) and 0.0002% methylene blue in reverse osmosis-distilled water. Zebrafish larvae were obtained from heterozygote crosses of our CRISPR-generated *arxa*, *eef1a2*, *gabrb3*, *scn1lab*, *strada*, *stxbp1b*, and *aldh7a1* lines (Griffin et al., 2021) in addition to the *scn1lab* line (*scn1lab* ^*s552*^) ENU-generated line originally provided by the laboratory of Dr. Herwig Baier. These *scn1lab* lines are considered phenotypically equivalent (Griffin et al., 2021). Although the CRISPR *scn1lab* mutant larvae were used for comorbid phenotyping, we exclusively used the ENU-generated *scn1lab* ^*s552*^ line for our pharmacological experiments as it has been more extensively validated and characterized in the context of drug screening for Dravet syndrome (Baraban et al., 2013; Dinday and Baraban, 2015; Sourbron et al., 2016; Griffin et al., 2017; Eimon et al., 2018; Sourbron et al., 2019; Whyte-Fagundes et al., 2024). All zebrafish lines used here were F5 generation or later. Larval zebrafish are sexually indistinguishable between 5-7 dpf.

### Drugs

Clemizole (ApexBio*), stiripentol (Sigma Aldrich) and valproic acid (Sigma Aldrich) were stored at −20 °C as 10 mM stocks until needed (*Tocris supply was toxic).

### Behavior assays

Larval behavior was monitored at room temperature (21–22 °C) with automated locomotion detection using a DanioVision system running EthoVision XT 11 software (DanioVision, Noldus Information Technology). The detection settings were as follows: method; DanioVision, sensitivity; 110, video pixel smoothing; low, track noise reduction; on, subject contour; 1 pixel (contour dilation, erode first then dilate), subject size; 4-4065. For each zebrafish line, experiments were performed with at least 3 different clutches and *post hoc* genotyping was done using protocols previously published (Griffin et al., 2021). As genotypes were determined after behavioral testing, sample sizes between wild-type and mutant groups varied. Nevertheless, group sizes were consistent with established standards for statistical rigor in zebrafish behavioral studies (Habjan et al., 2024).

#### Startle assay

To assess unconditioned startle responses, 6 or 7 dpf larvae were first moved from their home incubator to the experiment room and left undisturbed for at least 10 min. The larvae were individually transferred to a 96-well microplate, which was then placed in the DanioVision observation chamber and left for 15 min in the dark to allow acclimation. Each well contained ∼150 µL of embryo medium. *Light startle assay:* Baseline recordings were taken for 15 min. This was followed by three-2 s flashes (100% intensity), with intervals of 2 s dark. Activity was recorded during this period and for another 15 min (post-startle response). *Tap startle assay:* Baseline recordings were taken for 15 min. This was followed by 3-taps (level 8, highest), with 2 s intervals between taps. Activity was recorded during this period and for 15 min post-startle then larvae were lysed for genotyping.

#### Light-dark preference assay

We designed a 12-laned rectangular plate (8.6 cm length x 12.7 cm width x 1.4 cm height) with an equally sized ‘dark’ and ‘light’ arena (manufactured by Maze Engineers). The dark arena (walls and floor) was made with a black IR translucent, white light impermeant material while the light arena was clear and allowed both white and IR light. Light-dark tests were conducted in the DanioVision chamber with white (intensity: 20 or 100%) and IR light radiating from the floor of the chamber for the entirety of the experiment.

Six dpf larvae were first moved from their home incubator to the experiment room and left undisturbed for at least 10 min. The lanes were filled with 3 mL egg water and larvae were individually placed at the light-dark junction of the device using a transfer pipette. With the EthoVision program loaded, the plate was placed in the DanioVision chamber and acquisition window was selected so the program could accurately identify the larvae (1–2 min). Then larval behavior was recorded for 5–10 min and the total time and distance spent by the larvae in both arenas were quantified. For experiments using larvae from heterozygote in-crosses, genotyping was performed immediately following experimentation. A subset of the *scn1lab* larvae were subjected to phenylthiourea (PTU) treatment before the preference test. Specifically, between 1-4 dpf *scn1lab* embryos/larvae were treated with 0.003% PTU in 100 mm petri dishes and at 6 dpf scototaxis behavior was quantified.

### Pharmacology

Since the *scn1lab* mutants uniquely combined established clinical predictive validity (Griffin et al., 2018), high-throughput suitability facilitated by pigmentation-based identification (Baraban et al., 2013) and the most pronounced comorbid-related behavioral phenotypes observed across our assays, it was selected as the primary model for pharmacological testing.

*Startle*: *Scn1lab* mutant zebrafish (6 dpf) were identified by their dark appearance and each larva was individually transferred to a single well of a 96-well microplate containing 100 µL of embryo medium. Subsequently, 100 µL of drug solution (either 50 µM valproic acid, stiripentol, clemizole, or 0.1% DMSO (vehicle control)) was added to the corresponding wells, yielding a final drug concentration of 25 µM in each well. The plate was positioned in the observation chamber, left undisturbed for 15 min and then the light or tap startle assay was performed.

*Preference*: *Scn1lab* mutants (6 dpf) were treated with 25 µM valproic acid, stiripentol, clemizole or DMSO control in a 100 mm petri dish for 30 min. Following transfer to the center of the light-dark device, larval movement and arena preference were monitored for 5 min in the DanioVision chamber.

### Statistical analysis

The results were analyzed using the GraphPad Prism 10 software (San Diego, CA) and are plotted as mean ± standard error of mean. Normality was assessed using appropriate tests in GraphPad Prism. Depending on the experimental design, statistical significance was determined using two-way repeated measures (RM) ANOVA, followed by either Fisher’s least significant difference (LSD), Dunnett’s or Sidak’s multiple comparisons test. A two-tailed Mann-Whitney *U* test was used for two-group nonparametric comparisons. Asterisks denote significance levels: *p < 0.05; **p < 0.01; ***p < 0.001; ****p < 0.0001.

## Results

**FIGURE 1:**
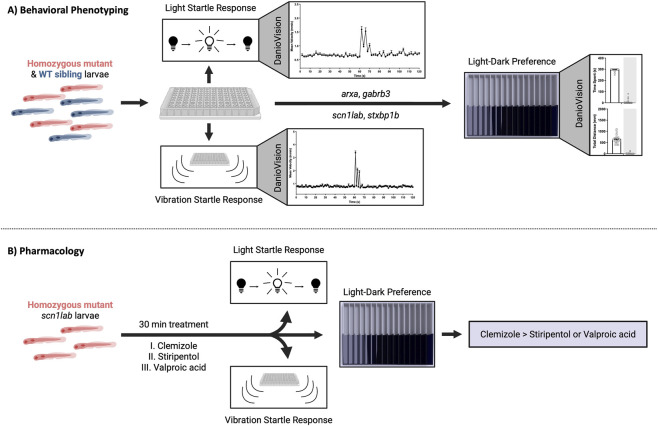
Behavioral phenotyping and pharmacological testing workflow in larval zebrafish. **(A)** Larval zebrafish lines (6 dpf) were subjected to light-evoked and vibration-induced startle assays in a 96-well format using DanioVision tracking, and a subset was subsequently assessed in a light-dark preference assay. **(B)** Homozygous *scn1lab* larvae were treated for 30 min with clemizole, stiripentol, or valproic acid, followed by startle and light-dark preference assays to assess behavioral rescue. Startle plots show mean velocity responses of WT (TL) larvae to light (three on-off pulses) and vibration (three taps) stimuli, with stimulus onset at 60 s. Light-dark preference plots show WT behavior under 20% light intensity, quantified by time spent (s) and total distance traveled (mm) in light versus dark (shaded gray) arenas. Data are presented as mean ± SEM (n = 60–70). Created with BioRender.

To characterize epilepsy-associated comorbid phenotypes in developing zebrafish, we established an integrated framework combining behavioral assessment with pharmacological rescue (Figures 1A,B). We first evaluated behavior using visual and tap startle assays. Normal startle responses in larvae are mediated by reticulospinal pathways that connect sensory neurons with spinal cord motor neurons (Koch, 1999). These responses manifest as rapid axial muscle contractions, which influence locomotor activity and play a critical role in enabling the organism to evade predators or avoid injury. Deviations from this typical response pattern can give insight into motor function and deficits in basic forms of learning (Kimmel et al., 1974; Colwill and Creton, 2011; Roberts et al., 2013). Visual startle responses in zebrafish larvae arise by 3 dpf (Easter and Nicola, 1996), with abrupt responses to vibrational/acoustic stimuli quantifiable after 5 dpf (Best and Alderton, 2008; Faria et al., 2019). Since seizure events have generally been measured in larval zebrafish models between 5-7 dpf (Afrikanova et al., 2013; Baraban, 2013; Dinday and Baraban, 2015; Griffin et al., 2021), we restricted our phenotyping for this study within this time frame as this period adequately overlaps with the development of startle behavior. We confirmed these behaviors in Tupfel Long (TL) zebrafish, which served as the wildtype (WT) background strain for creating the CRISPR epilepsy lines (Griffin et al., 2021). The results are captured in Figure 1A, where WT larvae exhibited robust visual and tap responses, with apparent habituation by the second and third stimuli presentation, respectively.

### Line-specific variations in startle response phenotypes

**FIGURE 2:**
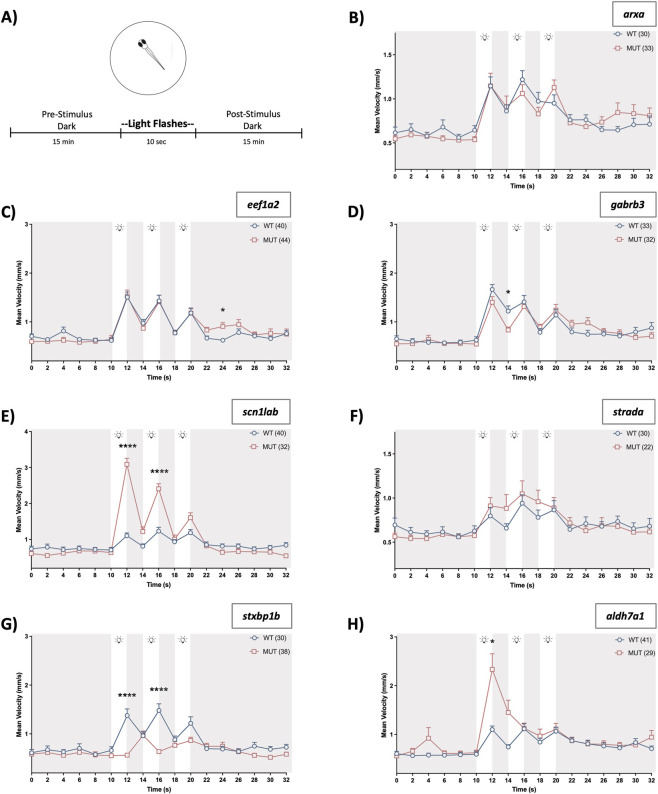
Light startle response. **(A)** Schematic highlighting that after acclimation, plated larvae are recorded for 15 min before and after light stimuli exposure (three flashes). **(B–H)** Plots depict larval movement assessed via mean velocity (mm/s) measurements. Time = 0 is 14:48 (min:s) after the beginning of the recording. Gray shaded areas capture time periods where 6 dpf larvae are in the dark and unshaded regions with light bulbs represent periods of flash stimuli. Data are shown as mean ± SEM. Post hoc Sidak’s multiple comparison test *p < 0.05 and ****p < 0.0001.

Heterozygote breeders for the six epileptic lines were in-crossed and at 6 dpf, larval movement was quantified in light startle assays using mean velocity measurements (Figures 2A–H). Two-way ANOVA analyses revealed that LOF mutations in *arxa*, *eef1a2* or *strada* did not alter startle response at 6 dpf. However, *gabrb3* mutants displayed a blunted response compared to WT (Figure 2D, two-way RM ANOVA, *F(*_16, 1008_) = 30.91, *p* < 0.0001 for time; *F* (_1, 63_) = 0.39, *p* = 0.54 for genotype; and *F* (_16, 1008_) = 2.22, *p* = 0.004 for time × genotype interaction). The *scn1lab* mutant larvae had a significantly greater visual startle response than WT siblings as shown by the higher mean velocities achieved when each stimulus was present (Figure 2E, two-way RM ANOVA, *F* (_8.689, 608.2_) = 61.84, *p* < 0.0001 for time; *F* (_1, 70_) = 7.73, *p* = 0.007 for genotype; and *F* (_8.689, 608.2_) = 28.62, *p* < 0.0001 for time × genotype interaction). An earlier report showed that *stxbp1b* mutants exhibit reduced locomotor activity after a sudden darkness (Grone et al., 2016) and here, we also saw an attenuated startle response in these larvae when exposed to a series of light flashes after being acclimated to the dark environment (Figure 2G, two-way RM ANOVA, *F* (_9.430, 622.4_) = 13.91, *p* < 0.0001 for time; *F* (_1, 66_) = 12.47, *p* = 0.0008 for genotype factor; and *F* (_9.430, 622.4_) = 8.84, *p* < 0.0001 for time × genotype interaction). Although *aldh7a1* mutants do not have spontaneous seizures at 6 dpf (Griffin et al., 2021), they emerge later in development at ∼10 dpf (Pena et al., 2017; Zabinyakov et al., 2017) and capture many facets of pyridoxine-dependent epilepsy. Given their relevance as a validated model of a severe developmental and epileptic encephalopathy, we also investigated their behaviors under this experimental paradigm. Similar to *scn1lab* mutants, *aldh7a1* larvae have a significantly heightened response to light flashes (Figure 2H, two-way RM ANOVA, *F* (_5.687, 386.7_) = 19.34, *p* < 0.0001 for time; *F* (_1, 68_) = 9.23, *p* = 0.003 for genotype; and *F* (_5.687, 386.7_) = 6.54, *p* < 0.0001 for time × genotype interaction).

**FIGURE 3:**
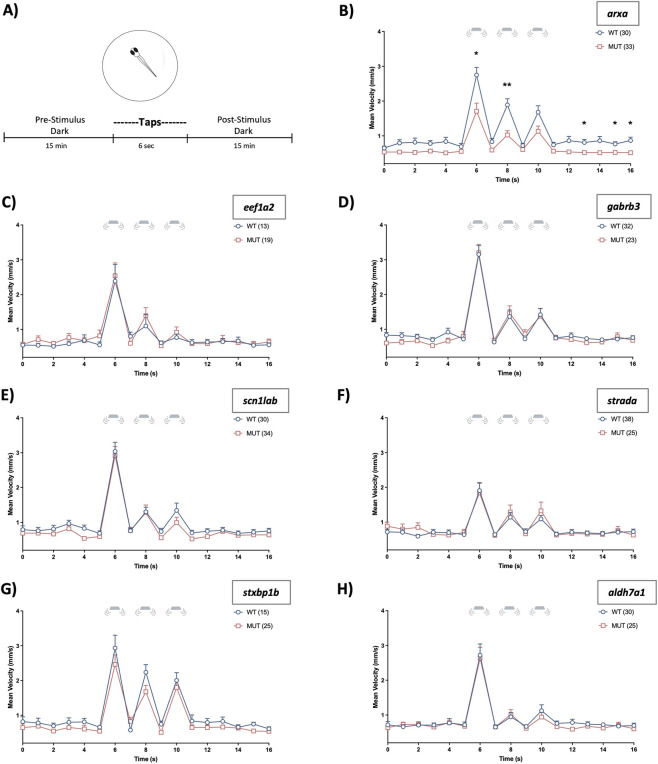
Tap startle response. **(A)** Schematic highlighting that after acclimation, plated 6 dpf larvae are recorded for 15 min before and after tap/vibrational stimuli exposure (three taps). **(B–H)** Plots depict larval movement assessed via mean velocity (mm/s) measurements. Time = 0 is 14:54 (min:s) after the beginning of the recording. Vibrating microplate icon signifies periods where tap stimuli is present. Data are shown as mean ± SEM. Post hoc Sidak’s multiple comparison test *p < 0.05 and **p < 0.01.

Next, we evaluated these same epileptic zebrafish lines following presentation of a tap stimuli series (Figures 3A–H). Although mutant *arxa* larvae showed no visual startle impairments, they did exhibit a diminished vibrational startle response (Figure 3B, two-way RM ANOVA, *F* (_4.502, 274.6_) = 43.42, *p* < 0.0001 for time; *F* (_1, 61_) = 37.36, *p* < 0.0001 for genotype; and *F* (_4.502, 274.6_) = 3.63, *p* = 0.0047 for time × genotype interaction). Post-stimulus hypoactivity was consistent with the reduced baseline movement observed during earlier characterization of this line (Griffin et al., 2021). At this developmental stage, the remaining epileptic mutants (*eef1a2*, *gabrb3*, *scn1lab*, *strada*, *stxbp1b*) in addition to the *aldh7a1* mutants had normal tap-evoked movement, with a similar pattern of stimuli habituation as their WT counterparts (Figures 3C–H).

### Decoupling of startle and seizure behaviors in *scn1lab* mutants

**FIGURE 4:**
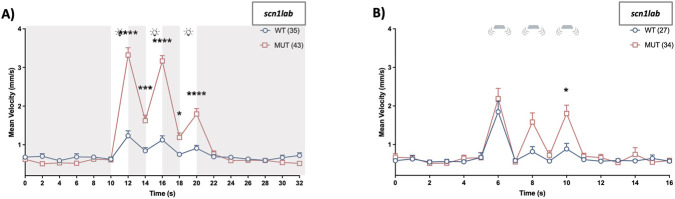
Heightened startle response persists to 7 dpf in *scn1lab* mutants. *Scn1lab* ^*+/−*^ breeders were crossed and on day 7, clutches were exposed to the **(A)** light and **(B)** tap startle assay. Startle responses were quantified via mean velocity (mm/s). Data are shown as mean ± SEM. Post hoc Sidak’s multiple comparison test *p < 0.05, ***p < 0.001 and ****p < 0.0001.

Larval *scn1lab* seizure occurrence has been studied extensively across numerous developmental stages and there is a noted decrease in severe events with increasing age (Baraban et al., 2013; Griffin et al., 2018). We next investigated whether the aberrant startle response observed in this line was sustained at later developmental stages. To do this we also subjected 7 dpf *scn1lab* mutant larvae to both light and tap stimuli and tracked their corresponding movement changes. We found a similar pattern of exaggerated startle when larvae were exposed to the light flash series (Figure 4A, two-way RM ANOVA, *F* (_6.392, 485.8_) = 94.38, *p* < 0.0001 for time; *F* (_1, 76_) = 51.17, *p* < 0.0001 for genotype; and *F* (_6.392, 485.8_) = 43.26, *p* < 0.0001 for time × genotype interaction). Interestingly, unlike our findings at 6 dpf, 7 dpf *scn1lab* mutants also showed abnormalities in their tap startle response. Notably, they show reduced habituation with repeated stimuli presentation unlike 6 dpf WT siblings (Figure 4B, two-way RM ANOVA, *F* (_4.787, 282.5_) = 24.61, *p* < 0.0001 for time; *F* (_1, 59_) = 4.65, *p* = 0.035 for genotype; and *F* (_4.787, 282.5_) = 3.20, *p* = 0.009 for time × genotype interaction). Hyperactivity can be used as a surrogate marker of seizure behavior in zebrafish and rodent models (Baraban et al., 2005; Coppola and Moshé, 2012; Gschwind et al., 2023; Whyte-Fagundes et al., 2025). In addition to increased locomotion, both genetic- and chemically-induced zebrafish seizure models commonly display whirlpool-like circling (previously classified as stage II) and clonus-like convulsions followed by loss of posture (stage III) (Baraban et al., 2005). Visual analysis indicated that neither the light nor tap stimuli elicited behaviors consistent with the patterns of stage II or III behavioral seizure events.

### *Scn1lab* and *stxbp1b* mutants display preference and exploratory abnormalities

Light-dark preference assays are commonly employed to assess novel environment exploration, locomotion and anxiety-like traits in preclinical studies. This assay capitalizes on the innate tendencies of animals, like zebrafish, to spend more or less time exploring certain areas of a given environment and has been validated using anxiolytic agents (Crawley, 1981; Costall et al., 1989; Bourin and Hascoët, 2003; Steenbergen et al., 2011). Larval zebrafish exhibit dark avoidance which eventually switches to a dark preference in adults (Dahlén et al., 2019). This preference change is postulated to give the more pigmented adult zebrafish an advantage in avoiding predators (Maximino et al., 2007). The degree of this avoidance can vary with experimental conditions, including light intensity (Córdova et al., 2016), therefore we first examined how white light intensity in the DanioVision chamber would affect the innate response of WT TL larvae (background) before evaluating epilepsy mutants. Our custom 12-laned device had light and dark arenas, the latter achieved with black IR translucent material on the walls and floors. Six dpf larvae were placed at the junction of the arenas and then their movements were monitored in the device for 10 min under conditions of low (20%) and high illumination (100%). Larvae exhibited a comparably strong positive phototactic response under both lighting conditions (Supplementary Figure S1). Consequently, the lower light intensity was selected for subsequent preference characterization in our zebrafish lines.

**FIGURE 5:**
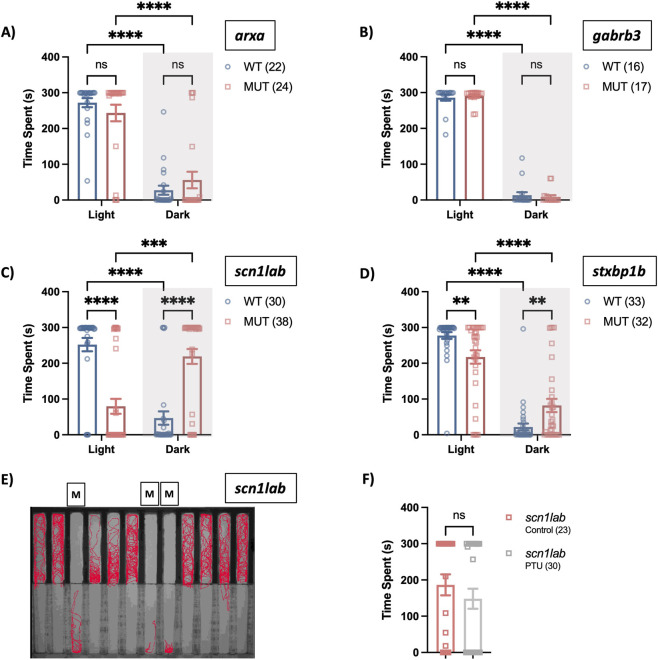
Scototaxis behavior in epilepsy lines. **(A–D)** *Arxa*, *gabrb3*, *scn1lab* and *stxbp1b* lines were subjected to the light-dark preference assay. Breeders were in-crossed and on day 6, larvae were transferred into the center of the 12-lane custom preference chamber. After 2 min in the DanioVision, time spent (s) in the dark versus light arena was measured for 5 min. **(E)** Representative figure showing cumulative *scn1lab* larval movement as indicated by red traces. Mutants are highlighted by label “M”. **(F)** Clutches generated from *scn1lab* were treated with PTU from 1-4 dpf. Light-dark preference behavior was assessed at 6 dpf using the device (via time-spent in light arena). Data displayed as mean ± SEM. Statistical analyses: **(A–D)** Two-way ANOVA with multiple comparison Fisher’s LSD test **p < 0.01, ***p < 0.001 and ****p < 0.0001. F) Two-tailed Mann-Whitney *U* test, *p < 0.05.

We restricted our preference analyses to the four lines with prominent startle defects, specifically, *arxa*, *gabrb3*, *stxbp1b* and *scn1lab*. When given a choice between light and dark arenas, *arxa* and *gabrb3* mutants spent more time in the light, showing a preference similar to WT at 6 dpf (Figures 5A,B). In contrast, *scn1lab* mutants spent most of their time by the walls in the dark arena (Figure 5C, two-way RM ANOVA, *F*_(1, 66)_ = 1.32, *p* = 0.2553 for arena; *F*_(1, 66)_ = 0.069, *p* = 0.793 for genotype; and *F*_(1, 66)_ = 35.98, *p* < 0.0001 for arena × genotype). This observed thigmotaxis was consistent with previous reports that utilized a traditional round well experimental design (Grone et al., 2017). *Stxbp1b* mutants spent most of their time in the light arena, though the time spent in the dark exceeded that observed in WT (Figure 5D, *F*_(1, 63)_ = 89.3, *p* < 0.0001 for arena; *F*_(1, 63)_ = 1.27, *p* = 0.263 for genotype; and *F*_(1, 63)_ = 8.45, *p* = 0.005 for arena × genotype). Figure 5E shows a representative locomotor trace (red lines) indicating that *scn1lab* mutants preferentially occupied the dark zone (bottom half of plate) and predominantly remained close to the arena walls, consistent with reduced exploratory behavior.

A complete reversal in the light-dark preference of *scn1lab* mutant larvae was a surprising finding. Given that late-stage dark preference in zebrafish has been associated with increased pigmentation, we examined whether hyperpigmentation in *scn1lab* mutants contributes to this phenotype. Starting at 1 dpf, *scn1lab* mutant larvae were treated with 0.003% PTU daily until day 5, then on day 6 we repeated the light-dark protocol. Using a two-tailed Mann-Whitney *U* test, we found that the PTU treatment did not increase the time spent by the *scn1lab* mutants in the light arena (Figure 5F, *U* = 253, *p* = 0.088). It is important to note that LOF *stxbp1b* also tend to have increased pigmentation (though not to the degree of LOF *scn1lab* zebrafish, (Grone et al., 2016)), thus their light preference further counters the role of pigmentation as the primary driver of dark preference in *scn1lab* mutants. In addition to time spent, we also quantified the total distance traveled in each arena, which followed similar trends across the different lines (Supplementary Figure S2).

### Clemizole reduces comorbid behavioral phenotypes in *scn1lab* mutants

**FIGURE 6:**
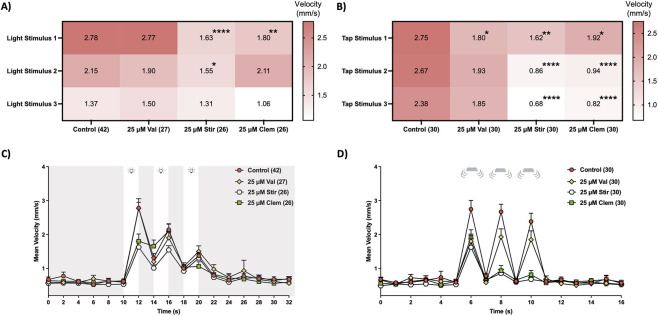
Clemizole and stiripentol alleviates light and tap startle abnormalities in Dravet zebrafish model. Six dpf *scn1lab* mutant larvae (identified by black pigmentation) were transferred to a 96-well microplate and treated with 25 µM valproic acid (Val), 25 µM stiripentol (Stir) or 25 µM clemizole (Clem) for 30 min prior to initiation of light or startle stimuli. Heat maps summarize the effects of drugs on mean velocity movements in the presence of the **(A)** three light flashes and **(B)** three taps. Line graphs illustrate the effects of the drugs before, during and after **(C)** light and **(D)** tap stimuli. Statistical significance highlighted in heat maps are excluded for visual clarity in **(C,D)**. Plots are represented as mean ± SEM. Post hoc Dunnett’s multiple comparison test *p < 0.05, **p < 0.01 and ****p < 0.0001.

**FIGURE 7:**
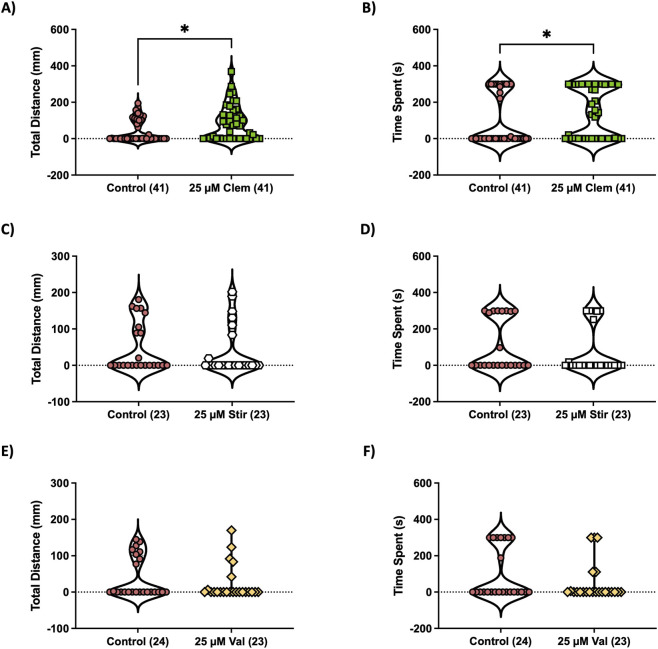
Clemizole improves exploratory behavior in Dravet zebrafish model. Prior to light-dark assay, we treated 6 dpf *scn1lab* mutant larvae with 25 µM valproic acid, 25 µM stiripentol or 25 µM clemizole for 30 min in a 100 mm petri dish. Fish were transferred to the center of the light-dark preference device and once in the DanioVision, total distance (mm) and time spent (s) in the light arena were assessed **(A-F)**. Two-tailed Mann-Whitney *U* test, *p < 0.05.

Of the zebrafish lines incorporated into this study, *scn1lab* mutants are the only to have undergone extensive antiseizure drug screening (Dinday and Baraban, 2015; Griffin et al., 2017; Griffin et al., 2020; Whyte-Fagundes et al., 2024). Serotonin modulating drugs, such as clemizole, lorcaserin and fenfluramine, have shown striking efficacy in reducing seizure activity (Dinday and Baraban, 2015; Sourbron et al., 2016; Griffin et al., 2017). We investigated whether clemizole (a novel therapy currently in Phase III clinical trials; https://www.clinicaltrials.gov/study/NCT04462770 and https://www.clinicaltrials.gov/study/NCT05066217), alongside two clinically relevant antiseizure medications representing distinct therapeutic strategies in Dravet syndrome, could also reduce the various abnormal behavioral phenotypes observed in our assays. We selected valproic acid as a first-line treatment and stiripentol as a syndrome-specific adjunctive therapy (Vasquez and Wirrell, 2025). While multiple antiseizure medications are used clinically, this focused selection prioritizes mechanistic and translational insight over breadth, particularly given evidence that current therapies often fail to address non-seizure comorbidities. We found that unlike valproic acid, 25 µM stiripentol and clemizole effectively reduced both aberrant light startle responses (Figure 6A, two-way RM ANOVA, *F*_(3, 117)_ = 3.86, *p* = 0.0112 for drug; *F*_(6.605, 772.7)_ = 91.86, *p* < 0.0001 for time; and *F*_(19.81, 772.7)_ = 2.98, *p* < 0.0001 for time × drug interaction) and tap startle responses (Figure 6B, two-way RM ANOVA, *F*_(3, 116)_ = 8.44, *p* < 0.0001 for drug; *F*_(19.59, 2273)_ = 24.34, *p* < 0.0001 for time; and *F*_(58.78, 2273)_ = 3.13, *p* < 0.0001 for time × drug interaction) in *scn1lab* mutant larvae. Figures 6C,D show the complete time-course plots that support the statistically significant effects reported in Figures 6A,B. Clemizole was the only drug to significantly increase larval exploration (i.e., increasing the time spent and total distance moved) by *scn1lab* mutants in the light arena in our preference assay (Figures 7A–F, A: *U* = 585, *p* = 0.013; B: *U* = 628, *p* = 0.037).

## Discussion

Researchers are increasingly using larval and adult zebrafish as models for a wide range of human diseases and disorders, such as cancer, cardiovascular and bone diseases, neurodegenerative conditions, and epilepsy (Adhish and Manjubala, 2023; Angom and Nakka, 2024). Given their growing utilization and recent designation as a New Approach Methodology (NAM) organism (Tanguay, 2025), there is a critical need to assess whether they adequately recapitulate the complexity of clinically observed symptoms. For instance, in the context of epilepsy, between 30% and 50% of patients struggle with comorbidities (Kanner, 2016). Genetic rodent models recapitulate several of these comorbidities (Warner et al., 2016; Jackson et al., 2017; Ricobaraza et al., 2019; Gonzalez-Sulser, 2020; Forrest et al., 2023). To be effective translational tools, genetic zebrafish epilepsy models should also reflect comorbidities. Advancements in gene-editing technologies have led to the development of zebrafish models that exhibit spontaneous, recurrent electrographic seizure activity (Grone et al., 2016; Pena et al., 2017; Scheldeman et al., 2017; Zabinyakov et al., 2017; Griffin et al., 2021; LaCoursiere et al., 2024; Lee et al., 2025), thus better mimicking the unprovoked nature of most seizures seen clinically. Epilepsy comorbid phenotypes have been reported in *stxbp1b* and *scn1lab* mutant zebrafish (Grone et al., 2016; Grone et al., 2017). Here we further validate and broaden these results by examining several additional genetic zebrafish models to provide a more thorough assessment of behavioral comorbidities.

Sensory processing abnormalities are highly prevalent among children with epilepsy (van Campen et al., 2015). Similar sensory challenges are well documented in autism spectrum disorder and attention-deficit/hyperactivity disorder, which are common comorbidities of epilepsy (Dunn, 2001; Mangeot et al., 2001; Kern et al., 2006; Ludlow et al., 2014). In cases where epilepsy and autism spectrum disorder or attention-deficit/hyperactivity disorder co-occur, these impairments tend to be even more pronounced (van Campen et al., 2015). These outcomes are evident in developmental and epileptic encephalopathies such as Dravet, West, and Ohtahara syndromes, which are associated with several of the genes under investigation (O'Brien et al., 2019; Bertuccelli et al., 2021). We examined sensorimotor integration in our zebrafish epilepsy lines using visual and mechanosensory (tap) stimuli, where including both stimuli provided an opportunity to understand whether the genetic mutations led to more global sensorimotor defect or specific modality dysfunction. *Scn1lab* mutant larvae demonstrated markedly atypical startle responses, with amplified motor reactions to both light and tap stimuli, indicating broad sensory processing deficits. Other lines showed asymmetry in disruptions. For instance, *stxbp1b* and *gabrb3* mutants demonstrated selective hypo-responsiveness to visual stimuli, with intact mechanosensory responses compared to wild-type controls. Conversely, *arxa* mutants only showed weakened reactivity to taps but preserved light-induced responses. Interestingly, the coexistence of opposing sensory phenotypes across different zebrafish epilepsy models mirrors the sensory heterogeneity documented in autism spectrum disorder (Balasco et al., 2019), highlighting potential mechanistic overlap.

“Dark flash” hypo-responsiveness was previously reported in *stxbp1b* mutant larvae, with accompanying melanosome dispersion under illuminated conditions, resulting in a markedly darker pigmentation compared to WT controls (Grone et al., 2016). In larval zebrafish, light exposure typically triggers melanosome aggregation via ocular photoreception, producing a lighter appearance (Shiraki et al., 2010). Therefore, it has been speculated that the pigmentation phenotype in *stxbp1b* mutants reflects disrupted photic input processing, which may serve as a major driver of their attenuated startle reflex (Grone et al., 2016). *Scn1lab* mutants are typically even more darkly pigmented yet exhibit a robust, exaggerated responses to both light and tap stimuli and thus challenges this interpretation. Notably, *scn1lab* mutant larvae exhibit a higher seizure frequency than *stxbp1b* mutants (Griffin et al., 2021). This elevated seizure burden may contribute to heightened physiological stress, a factor associated with melanosome dispersion in zebrafish (Piato et al., 2011). Therefore, stress-related mechanisms rather than impaired photic processing may underlie the pigmentation differences observed in some models and warrant further investigation.

Genetic mouse models corresponding to the zebrafish lines exhibiting atypical startle responses during stimulus presentation also display increased anxiety-like traits. For example, when examined in elevated plus and light-dark tests, *Stxbp1* deficient mice display impaired exploratory behavior and heightened stereotypy (Kovacevic et al., 2018; Chen et al., 2020). Furthermore, adolescent *Scn1a* ^*+/−*^ mice show increased wall-hugging tendencies (Ricobaraza et al., 2019; Bahceci et al., 2020) and *Gabrb3* ^*−/−*^ mice also present with abnormal exploratory patterns and anxiety-linked risk assessment behaviors (Hashemi et al., 2007; DeLorey et al., 2008). Although historically more common in mammalian model systems, anxiety-related assays are gaining traction in zebrafish studies. The application of an open field 6-well assay enabled detection of strong “wall hugging” behavior in *scn1lab* mutant larvae (Grone et al., 2017). Here, we used a custom device to further assess both light-dark preference and wall-hugging in *arxa*, *gabrb3*, *scn1lab* and *stxbp1b* mutants. In line with previous reports, *scn1lab* mutant larvae spent most of their time along the periphery but also showed a surprising reversal in light-dark preference, further suggesting underlying deficits in neurosensory development and behavioral regulation. While *stxbp1b* zebrafish, like *arxa* and *gabrb3* mutants, preferred the light zone, they also exhibited increased locomotor activity in the dark arena compared to WT controls. Using time spent in the light zone as a proxy for anxiety, this implies that these fish exhibit reduced anxiety-like behavior. Considering clinical observations alongside findings from rodent models, this result was unexpected and emphasizes the need for careful interpretation across species. Notably, variations in zebrafish light-dark tests, such as initial placement and metrics used, can significantly influence outcomes. In our setup, larvae were positioned at the light-dark boundary, and behavior was quantified based on time and distance in each zone after a brief acclimation. Future experiments employing alternative paradigms and extended developmental timepoints will be essential to provide a more in-depth assessment of anxiety-like phenotypes.

Another key finding from our study is the differential drug response observed in *scn1lab* mutants. Clemizole and diazepam were previously shown to rescue the open field deficit in this model (Grone et al., 2017). Using our light-dark chamber and startle assays, we also demonstrate that clemizole increases exploratory behavior and improves markers of sensory deficits in *scn1lab* mutants. These findings further support its potential as a novel therapy for Dravet syndrome, with benefits extending beyond seizure control. Stiripentol, approved as an adjuvant treatment for Dravet syndrome, only ameliorated the former phenotype. The latter finding underscores the growing recognition that seizure-focused therapies may inadequately address broader cognitive or behavioral impairments. The dual effect of clemizole on both seizures and comorbid behaviors suggests drug discovery pipelines should prioritize agents with wide-ranging neurodevelopmental benefits. Zebrafish are well-suited for high-throughput screening, enabling efficient identification of lead compounds and supporting a shift from seizure-focused to syndrome-based approaches.

Our zebrafish genetic lines are associated with some of the most severe, previously termed ‘catastrophic,’ pediatric epilepsies (Howard and Baraban, 2017). Yet, in many cases, we found subtle or no phenotypic differences when we compared our mutant lines to WT siblings. Our behavioral analyses were intentionally conducted at 6-7 dpf, a developmental window in which zebrafish larvae exhibit robust and quantifiable locomotor and sensorimotor responses. Earlier stages (<5 dpf) are characterized by limited spontaneous swimming and underdeveloped behavioral repertoires, making them less suitable for assessing complex behaviors such as startle responses and exploratory preference. Conversely, later stages (>7 dpf) introduce confounds related to feeding requirements and increased variability in environmental interactions (Fero et al., 2011; Wilson, 2012). Importantly, our window aligns with prior high-throughput seizure phenotyping in these models (Baraban et al., 2013; Grone et al., 2016; Griffin et al., 2021), allowing for direct comparison between seizure activity and comorbid behavioral outcomes. Nonetheless, expanding the scope of phenotypic screening, including longitudinal assessments, may help reveal additional subclinical or context-dependent traits. We also acknowledge a limitation in that these zebrafish models and experimental conditions may not capture the polygenetic drivers and/or environmental cues that drive some comorbidities. Additionally, the behavioral and physiological readouts obtained from these zebrafish models should be interpreted as proxies for comorbid behavioral phenotypes rather than direct equivalents. We do hope that our future work will further address the translational relevance of these markers. Clemizole, currently in a Phase III clinical trial as an adjunctive therapy for Dravet syndrome (https://clinicaltrials.gov/study/NCT04462770), may serve as a useful benchmark in this regard; if ultimately approved, its effects on non-seizure symptoms could provide valuable insights into the power and limitations of zebrafish models.

## Data Availability

Original datasets are available in a publicly accessible repository. This data can be found here: https://scholarship.richmond.edu/biology-faculty-publications/342/.

## Supplementary material

SUPPLEMENTARY FIGURE S1 Effect of light intensity on light-dark preference patterns of zebrafish larvae. At 6 dpf, WT (TL) larvae were placed in the 12-lane custom light-dark preference chamber. Behavioral activity was tracked for 10 min (in 5 min time bins) to assess preference and quantified by assessing the time spent (s) in the light versus dark zones under low illumination (20% light intensity) and high illumination (100% light intensity). Data displayed as mean ± SEM. Multiple comparison Fisher’s LSD test **p* < 0.05 and *****p* < 0.0001.

SUPPLEMENTARY FIGURE S2 Movement patterns across light-dark arenas in epilepsy lines. **(A-D)** *Arxa*, *gabrb3*, *scn1lab* and *stxbp1b* lines were subjected to the light-dark preference assay. Breeders were in-crossed and on day 6, larvae were transferred into the center of the 12-lane custom preference chamber. After 2 min in the DanioVision, total distance moved (mm) in the dark versus light arena was measured for 5 min. Data displayed as mean ± SEM. Multiple comparison Fisher’s LSD test **p* < 0.05, ***p* < 0.01 and *****p* < 0.0001.
