# Supplementary figures and images for: Employing zebrafish to understand genetic drivers of epilepsy-related comorbid behaviors
Source: Front Pharmacol. 2026 May 4;17:1781517. doi: 10.3389/fphar.2026.1781517 (PMC13180867; doi:10.3389/fphar.2026.1781517)

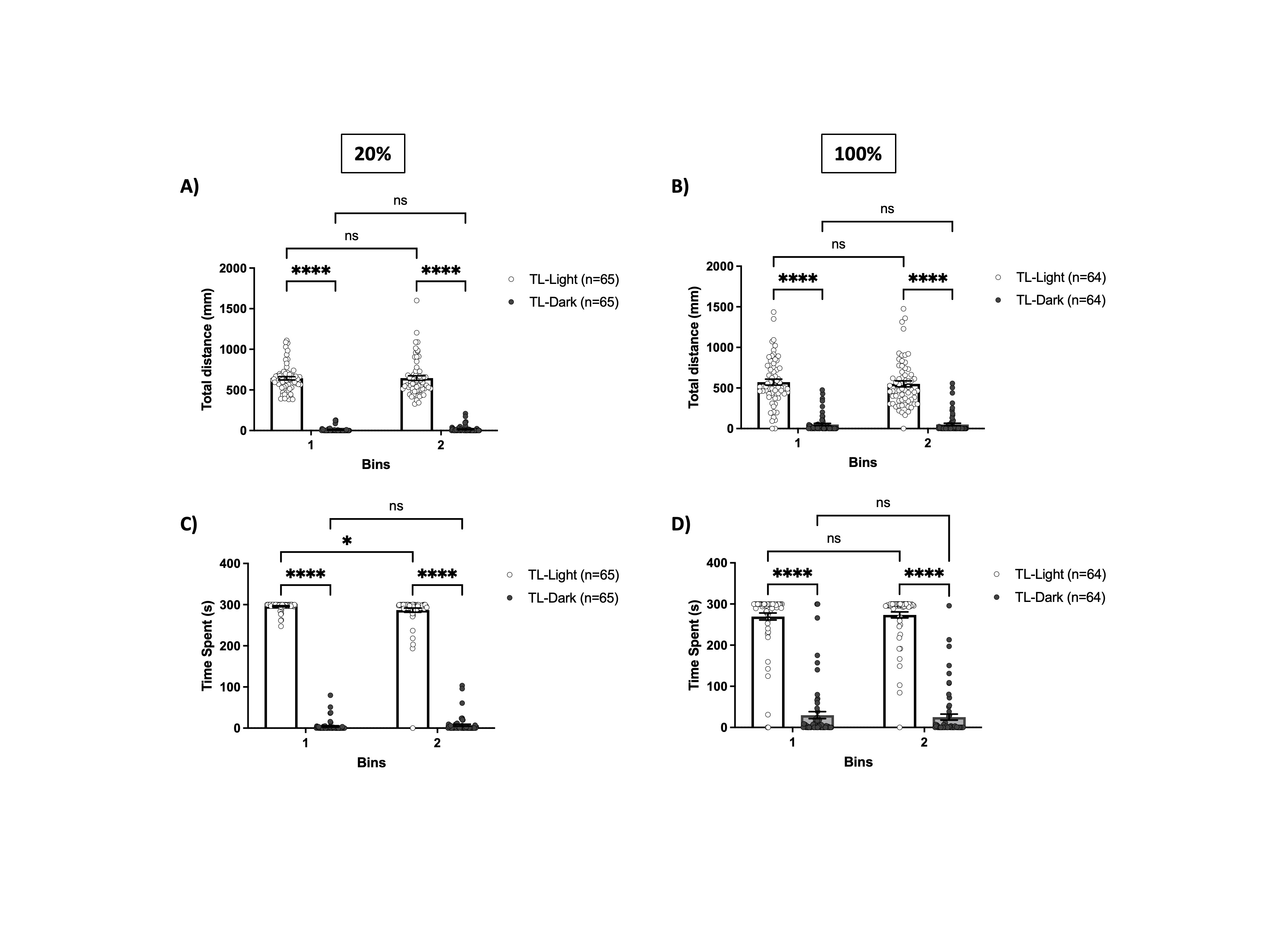

Supplement: Supplementary file 1 [file Image1.jpeg]

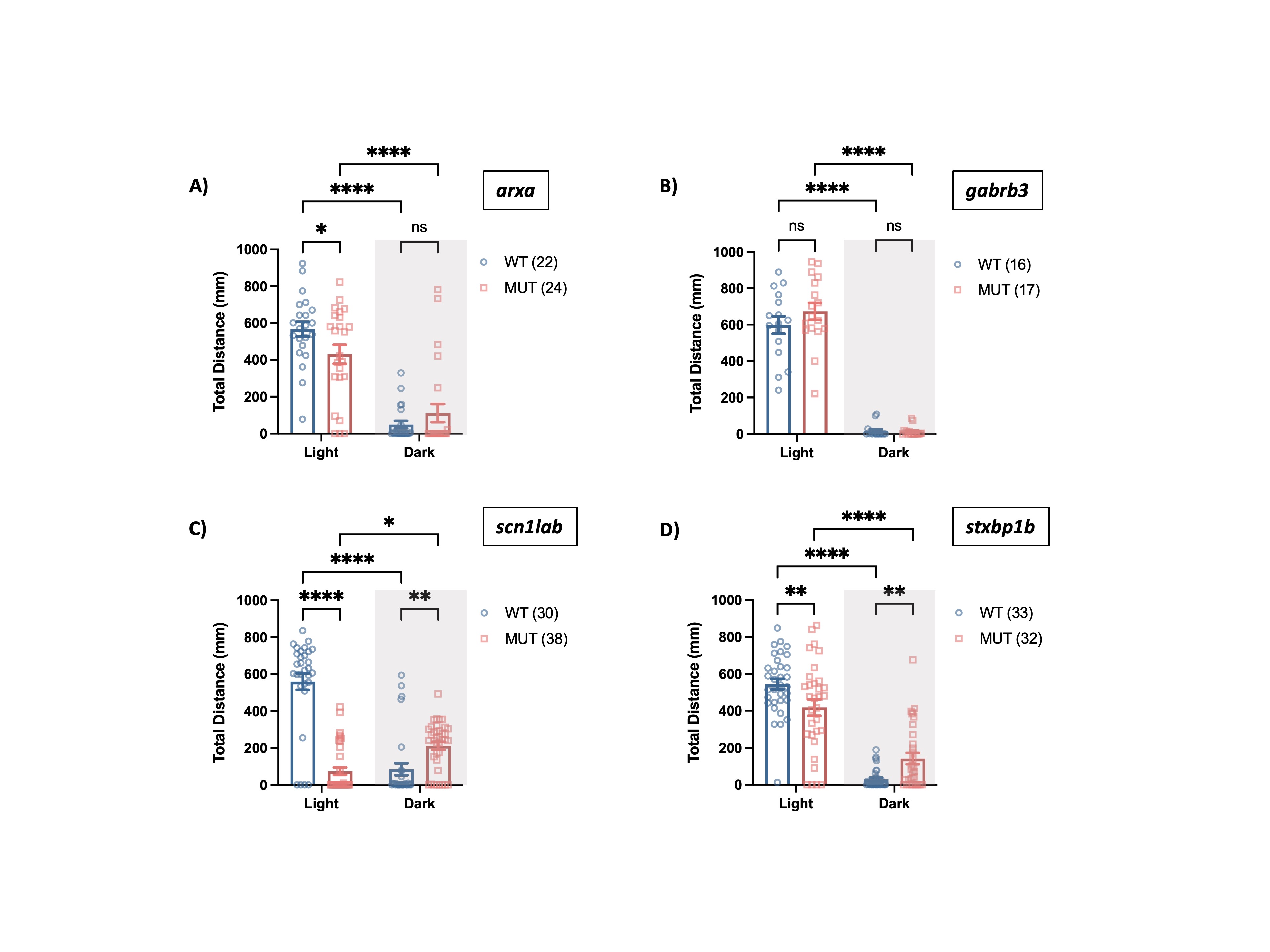

Supplement: Supplementary file 2 [file Image2.jpeg]
